# Temporal Convolutional Neural Networks for Radar Micro-Doppler Based Gait Recognition †

**DOI:** 10.3390/s21020381

**Published:** 2021-01-07

**Authors:** Pia Addabbo, Mario Luca Bernardi, Filippo Biondi, Marta Cimitile, Carmine Clemente, Danilo Orlando

**Affiliations:** 1 Science and Technology for Transportations Faculty, Università degli Studi “Giustino Fortunato”, Viale Raffale Delcogliano, 12, 82100 Benevento, Italy; p.addabbo@unifortunato.eu; 2 Department of Engineering, University of Sannio, Via Traiano, 1, 82100 Benevento, Italy; bernardi@unisannio.it; 3 Electromagnetic Laboratory, Engineering Faculty, Università degli Studi dell’Aquila, Piazzale E. Pontieri, Monteluco di Roio, 67100 L’Aquila, Italy; filippo.biondi@marina.difesa.it; 4 Unitelma Sapienza, Viale Regina Elena, 295, 00161 Rome, Italy; marta.cimitile@unitelmasapienza.it; 5 Center for Signal and Image Processing, Department of Electronic and Electrical Engineering, University of Strathclyde, Glasgow G1 1XW, UK; carmine.clemente@strath.ac.uk; 6 Engineering Faculty, Università degli Studi “Niccolò Cusano”, Via Don Carlo Gnocchi, 3, 00166 Rome, Italy

**Keywords:** deep learning, gait recognition, low-power radar, micro-Doppler, human ID

## Abstract

The capability of sensors to identify individuals in a specific scenario is a topic of high relevance for sensitive sectors such as public security. A traditional approach involves cameras; however, camera-based surveillance systems lack discretion and have high computational and storing requirements in order to perform human identification. Moreover, they are strongly influenced by external factors (e.g., light and weather). This paper proposes an approach based on a temporal convolutional deep neural networks classifier applied to radar micro-Doppler signatures in order to identify individuals. Both sensor and processing requirements ensure a low size weight and power profile, enabling large scale deployment of discrete human identification systems. The proposed approach is assessed on real data concerning 106 individuals. The results show good accuracy of the classifier (the best obtained accuracy is 0.89 with an F1-score of 0.885) and improved performance when compared to other standard approaches.

## 1. Introduction

A challenging and critical task in the video-surveillance domain is quick and accurate individual identification. Traditional approaches involving cameras only, namely, camera-based surveillance systems, lack discretion (privacy issues) and have high computational and storing requirements in order to perform human identification. Moreover, the performance of such systems depends on external factors (i.e., light and weather). For this reason, it would be highly desirable to integrate them with different kinds of sensors that can provide reliable performance also in adverse scenarios. These limitations can be exceeded by adopting radar sensors that require a smaller amount of data, are able to see through the walls, and are not affected by environmental conditions. Moreover, since radar sensors have low cost and low power consumption they represent a promising solution not only for future application in the surveillance context but also in other sectors (i.e., low Size Weight and Power radars are very diffused in the automotive industry). Basing on the above assumptions, several low power frequency-modulated continuous-wave (FMCW) radar algorithms for surveillance applications have been proposed in recent studies [1,2,3,4]. In Refs. [5,6], authors discuss the advantages of radar micro-Doppler (MD), highlighting how Doppler information generated by the movement of the target is useful for its identification and for the subsequent micro-motion analysis. The micro-Doppler classification capabilities are also confirmed in several other studies [7,8,9,10,11,12]. Since data produced by a FMCW data is particularly suitable for neural-networks processing, it is worthwhile investigating the adoption of Deep Learning (DL) algorithms for the gait-based human recognition using micro-Doppler signatures as features [13,14,15,16]. DL-based approaches extend classical machine learning ones using deeper neural networks that are capable of learning directly from more complex data, leading to better end-to-end classification and prediction performances. DL, taking inspiration by the way information is processed in biological nervous systems and their neurons, represent the data hierarchically, through several levels of abstraction corresponding to various artificial perceptrons [17]. For this reason, the DL approaches are based on deep neural networks composed of sets of hidden layers: in each step, the input data is transformed into a slightly more abstract and composite one. The hierarchical and conceptual representation of the layers is very useful to perform pattern classification, recognition, and feature learning.

In this paper, the temporal convolutional networks (TCNs) are used to identify individuals based on their gait dynamics. TCNs are a kind of deep neural networks with a convolution architecture design characterized by casualness and an output sequence of constant length [18]. Given their architecture, TCNs are particularly suitable to the gait recognition since in this context the causal relationships of the gait signal evolution can be learned. It is worth to highlight that the main contribution of this work is represented by the proposed TCN architecture which is composed of a two-level hierarchical attention layer stack as done in [19] for Recurrent Neural Netoworks (RNNs). Informally, a neural attention mechanism gives a neural network the capability to focus on a subset of its inputs (or characteristics).

This work builds upon a prior work published in [20] and extends the preliminary analysis to more complex scenarios with more individuals acquired in different environments. This allows us to verify the robustness, the scalability, and portability of the proposed methodology. Moreover, differently from [20], in this study the spectrograms are obtained using three different Hamming windows of 0.5, 1, and 2 milliseconds with 98% overlap. The 3 different window sizes will be used in different testing setups to assess the influence of the time-frequency resolution trade-off. Furthermore, the used radar presents a longer wavelength thus allowing for the identification of individuals at higher distances.

Our findings show that even if this reduces the discrimination capability of micro-Doppler signatures (smaller Doppler bands), the neural classifier is able to perform an efficient target recognition.

The assessment of the TCN classifier is performed on a relatively large dataset built at the University of Glasgow, including several walking sessions from 106 subjects (targets). The obtained results show the effectiveness of the proposed technique with respect to other baselines.

This paper is organized into five sections. Section 2 describes the related work. Section 3 shows the proposed methodology (respectively the gait MD features model and the TCN classifier are represented). Section 4 introduces and discusses the experiments performed to assess the proposed method. Finally, in Section 5 the conclusions are reported.

## 2. Related Work

The adoption of radars as sensors to perform human identification is largely discussed in the last years.

In particular, several approaches introduce machine learning algorithms to identify individuals from a set of features gathered from micro-Doppler radar [21,22]. These approaches show good performance: for example, in [22], Gaussian Mixture Models [23] are used to identify eight individuals, obtaining an accuracy greater than 90%.

More recently, some studies propose the combination of micro-Doppler data and deep learning algorithms for the gait-based human recognition [13,14,15,16]. However, the hierarchical structure of deep learning is more suitable to identify complicated patterns from raw data (i.e., images and signals) without any feature extraction [24,25]. According to this, in [13], a deep autoencoder is used to perform human gait recognition with micro-Doppler radar. In this study, the best classification rate (96.2%) is obtained when a bayesian optimization is performed to identify the suitable hyperparameters combination. Similarly, Ref. [15] proposes a Deep Convolutional Neural Network (CNN) [26] approach are used on micro-Doppler spectrograms achieving average accuracy between 97.1% and 68.9% on the base of the number of people involved in the experiment (from 4 to 20). CNN are also used in [16], where authors describe an approach to perform indoor gait based human recognition from micro-Doppler signatures extracted by a low-power radar device. The achieved classification error rate is of 24.70% on the validation set and 21.54% on the test set. Another CNN [26] based approach is proposed in [27] where human detection is performed by using a CNN [26] classifier on micro-Doppler spectrograms. The accuracy achieved for human detection is 97.6%.

The above studies are also discussed in [28], where authors introduce the inception architecture to human gait micro-Doppler features for the first time. The obtained accuracy rate in persons recognition usind a CNN classifier is around 96.9%. With respect to the above literature, this study proposes the adoption of a TCN classifier to identify individuals from the micro-Doppler data. The surrounding idea is that, given their casualness in the convolution architecture design, TCN is suitable to our context where the causal relationships of the gait signal evolution should be learned. Finally, this paper extends the study proposed in [20] by adding further details on the proposed approach generalizing the obtained results on a novel more challenging dataset.

## 3. The Proposed Methodology

### 3.1. Gait MD Feature Model

The Micro-Doppler (MD) effect induced by mechanical vibrating or rotating structures in a radar target is a very useful feature for target detection, classification, and recognition. In fact, while the Doppler frequency induced by the target body is constant, the MD due to the motions of target’s structures is a function of the listening time. Thus, the analysis of the time-varying Doppler signature in the joint time-frequency domain can provide precious information [29,30].

**Figure 1:**
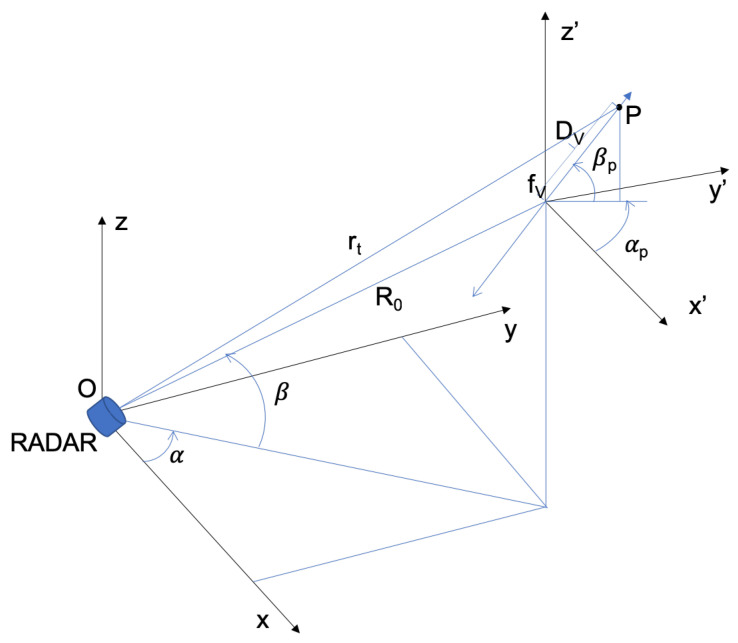
Observation geometry.

In Figure 1, the geometry used to analyse the micro-Doppler induced by a point-target *P*, vibrating with frequency $f_{v}$ at distances $R_{0}$ from the radar and $D_{v}$ from the center of coordinates $\left(x^{\prime },y^{\prime },z^{\prime }\right)$, is shown [30]. The list of used symbols and their meanings is reported in Table 1. Using a simplified model in the slow-time domain, the radar received signal can be expressed as

(1) $$s\left(t\right)=\rho e^{j\frac{4\pi R_{0}}{\lambda }}e^{j(2\pi f_{0}t+4\pi r\left(t\right)/\lambda )}$$

where:

- ρ is the backscattering coefficient;
- λ is the carrier wavelength;
- $r\left(t\right)=R_{0}+D_{v}\sin\left(\omega_{v}t\right)\cos\left(\beta \right)\cos\left(\alpha_{p}\right)$, with $\omega_{v}=2\pi f_{v}$, is the range function varying with time due to micro-motion.

By taking the derivative of the time-derivative of the second phase term, the micro-Doppler frequency induced by the vibration is

(2) $$f_{mD}\left(t\right)=\frac{D_{v}\omega_{v}}{\lambda }\cos\left(\beta \right)\cos\left(\alpha_{p}\right)\cos\left(\omega_{v}t\right).$$

**Figure 2:**
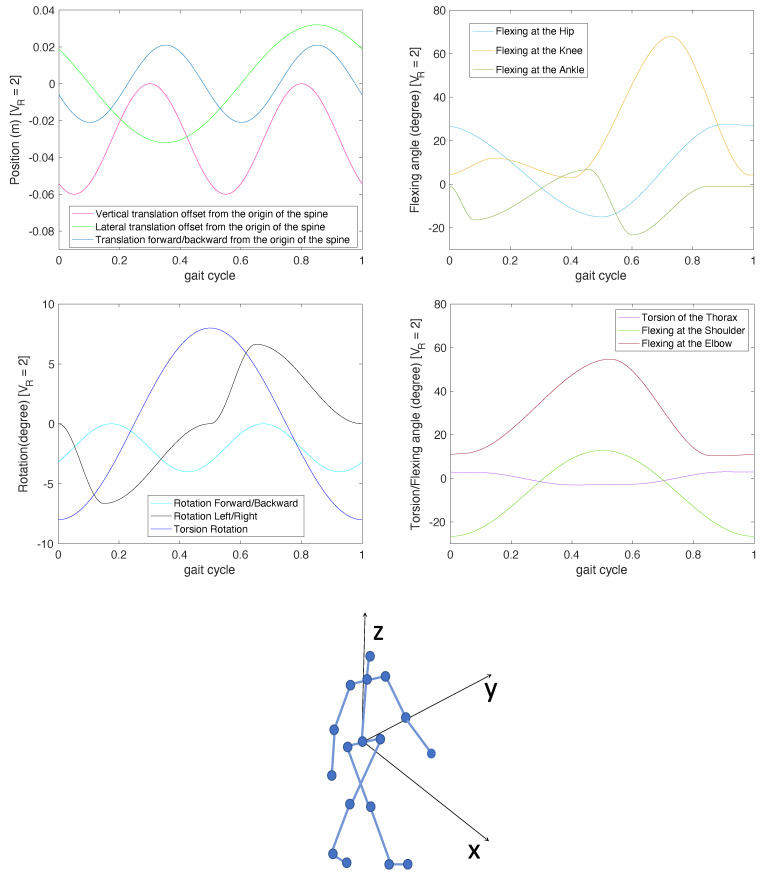
Human walking trajectories.

This simplified model could be generalized to more complex scenarios. However, it is worth noticing that an object or any structural object’s component may have oscillatory motion, which can be referred to as micro-motion, including any oscillatory motion in addition to the bulk motion of the object. For the case at hand, human articulated motion is composed by a superposition of different motions of the human body parts. A global human walk model based on empirical mathematical parameterizations has been derived in [31]. Specifically, the motion is described by 12 trajectories, 3 translations, and 14 rotations, five of which are duplicated for both sides of the body, as shown in Figure 2.

The spectrogram is the most common tool used for the representation of the micro-Doppler signatures. It is obtained through the calculation of the square module of the short-time Fourier transform (STFT) of the received signal

(3) $$\chi \left(\tau ,f\right)={\left|STFT\left(s\right)\right|}^{2}={\left|\int_{-\infty }^{+\infty }s\left(t\right)h\left(t-\tau \right)e^{-j2\pi ft}dt\right|}^{2},$$

where h(·) is the window function. In practice, the STFT is performed using the fast Fourier transform (FFT), so both the signal and the window function are discrete and quantized. Moreover, basically, the STFT can be interpreted as the Fourier transform of the “windowed” signal s(t)h(t−τ). The resolution of the STFT is determined by the window size and there is a trade-off between the time resolution and the frequency resolution: a larger window implies a higher-frequency resolution but a poorer time resolution. The Gabor transform is a typical short-time Fourier transform using Gaussian windowing and has the minimal product of the time resolution and the frequency resolution.

**Figure 3:**
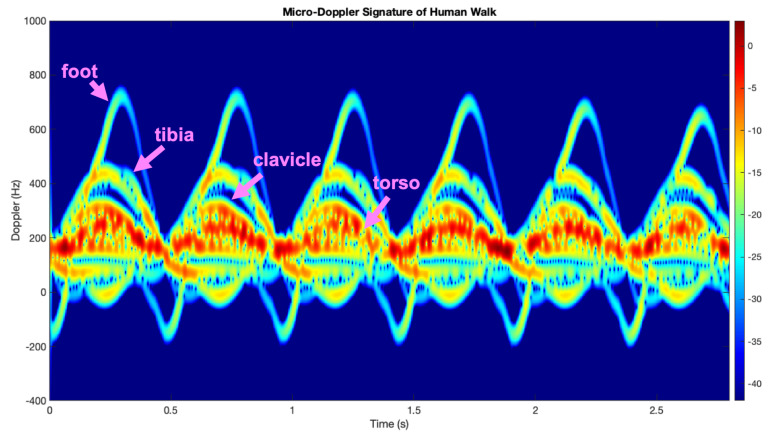
Micro-Doppler (MD) signature of Human Walk [29].

As an example, the micro-Doppler signature of the simulation for the translations and the rotations in one cycle of walking motion (i.e., from right heel strike to right heel strike) is shown in Figure 3. From figure’s inspection, it can be noticed that each forward swing of the leg produces large spikes and the movement of the torso which is the strongest component underneath the leg swings tends to have a slightly sawtooth shape because the speeding up and slowing down during the swing.

Finally, real radar measurements would present a strong clutter component due to background objects and not of interest moving objects. Stationary background objects can be easily suppressed by removing the zero Doppler component. Unwanted moving objects may also be filtered out thanks to the different ranges and speeds.

**Figure 4:**
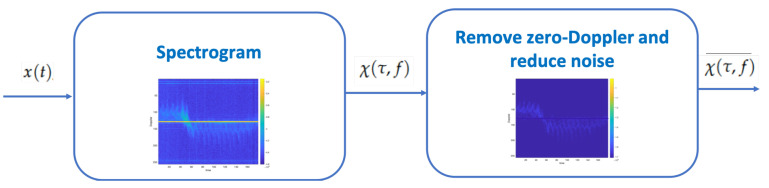
MD Feature extraction.

The main steps for a data analysis using a radar for gait MD feature extraction are shown in Figure 4. The first step consists in collecting data from the radar and processing it calculating the spectogram. In the next step, MD signatures are pre-processed through clutter and noise reduction.

**Figure 5:**
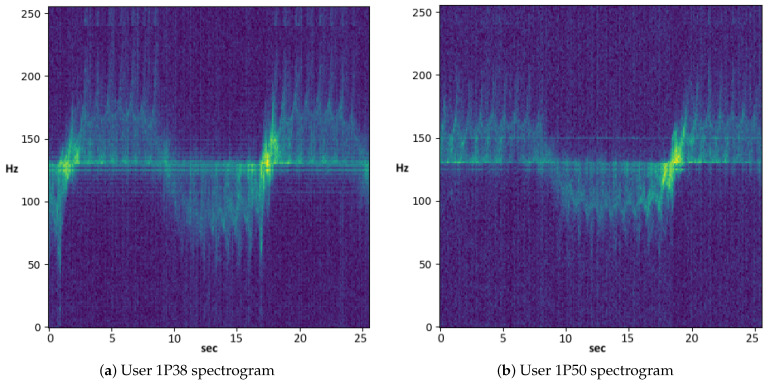
Spectrograms of two walkers.

Figure 5 shows the spectrograms of a 25.5 s window of two users given as input to the TCN network. As can be seen from the figure, identifying the distinctive features of the spectrogram that belong to a given user is not a simple task, surely for a human but even by means of classic machine learning approaches (e.g., decision trees or support vector machines). To learn the patterns and dynamics of the detected walkers, larger neural networks are needed, since they are capable of extracting complex features by recombining and processing them using larger numbers of layers. Our decision to use TCN is based on the fact that this kind of neural network is characterized by causalness in the convolution architecture design making it suitable to our classification problem where the relationships among the spectrogram sequences and the walking target should be learned. However, the proposed variant also includes a two-stage attention layer structure allowing to better capture both lower and higher dynamics that characterize micro-doppler signatures.

### 3.2. The TCN Classifier

**Figure 6:**
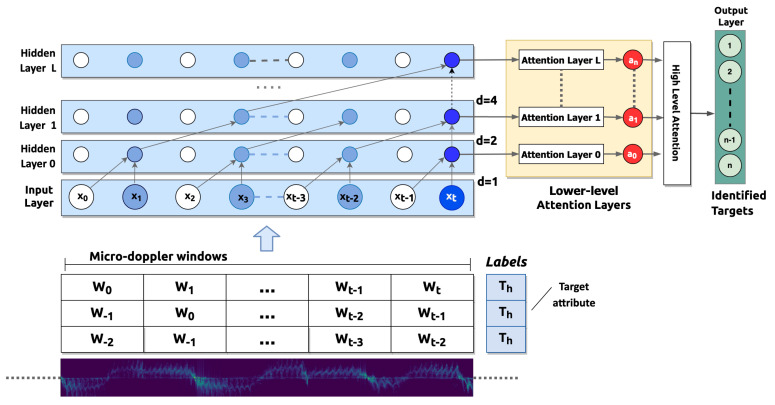
Temporal convolutional network (TCN) classifier architecture.

Figure 6 depicts the classification process realized in this study. The figure shows a TCN classifier trained by a set of micro-doppler time windows *W*. These windows are extracted from the spectrograms, like those shown in Figure 5, and used for neural networks training process. Each set of windows (represented by a row of the table in the lower part of the figure) is computed as a feature vector representing a single instance associated with a multinomial label Th, which specifies the person identity (target attribute). The spectrograms sliding windows are given as input to the network and are propagated through the layers of the TCN with a dilation factor that doubles on each layer.

In the training step, in order to perform validation, a 10-fold cross-validation is used [32]. Finally, the trained classifier is assessed on a the test set composed of data gathered from walking sessions never used before. The classifier is realized with a TCN architecture [18] that uses a 1D fully-convolutional network (FCN) architecture.

In the proposed architecture, three types of layers are considered: an input layer, a hidden layer, and an attention layer. The input layer is the neural network entry point and includes a node for each set of considered features at a given time.

The hidden layers are instead made of artificial neurons (they are also called “perceptrons”). The output of each neuron is computed as a weighted sum of its inputs and passed through an activation function or a soft-plus function. In the proposed architecture, a different number of hidden layers can be used: all the hidden layers have the same length as the input layer. However, a padding of length (kernel size-1) is added to enforce the layer length coherence and keep subsequent layers at the same length as the previous ones. This architecture allows us to ensure that at each evaluation, the output is obtained by considering only the current and the previous samples. Moreover, the architecture employs dilated convolutions that enable an exponentially large receptive field on the base of a dilation factor $d_{f}$ (a sort of fixed step) between every two adjacent filters. At the increase of the layer number, the dilation factor grows exponentially. However, when the kernel size is $k_{l}$, the data used at the lower layer is $(k_{l}-1)d$ and still grows exponentially at the increasing of the network layers. The classification is finally performed on the last sequential activation of the last layer (output layer) which synthesizes the information extracted from the complete input sequence into a single feature vector and produces the requested output.

Moreover, while this representation can be very reductive with respect to the high number of complex relationships, a hierarchical attention mechanism [19] is added across the network layers similarly to [33,34,35]. Attention layers model the relationships regardless of their distance in the input or output sequences.

Looking at Figure 6, for the TCN having *n* hidden layers, the weights matrix $L_{i}\in R^{K\times T}$ is defined as:

(4) $$L_{i}=\left[l_{1}^{i},...,l_{T}^{i}\right],$$

where *i* is the layer number containing the convolutional activations (with i=1,⋯,n), *K* is the filters’ number at each layer and *T* is the length of the window.

Moreover, we can define the layer attention weight $m_{i}\in R^{1\times T}$ as:

(5) $$m_{i}=\mathrm{softmax}\left(\tanh\left(w_{i}^{T}L_{i}\right)\right)$$

where $w_{i}\in R^{K\times 1}$ are the trainable parameter vectors. For the layer *i*, the corresponding set of convolutional activations is computed as $a_{i}\in R^{K\times 1}=f\left(L_{i}\beta_{i}^{T}\right)$ where f(·) is one activation function among ReLU, Mish and Swish [36] and $\beta_{i}$ are the weights of the attention layer. Finally, the convolutional activations $A\in \;R^{K\times n}=\left[a_{1},...,a_{i},...,a_{n}\right]$ of the hidden layers allow to compute the representation of the last sequence to ensure the final classification:

(6) $$\alpha =\mathrm{softmax}(\tanh\left(\omega^{T}A\right))$$

(7) $$y=f(A\alpha^{T})$$

where $\omega \in R^{K\times 1}$ and $\alpha \in R^{1\times K}$ are respectively the vector of weights and the output of the high-level attention layer, and $y\in R^{K\times 1}$ is the neural network final. Notice that, the batch normalization [37] is also added to improve the training of deep feed-forward neural networks. In the training step, we tested different combinations of architectural parameters (i.e., number of layers, batch size, optimization algorithm, and activation functions) to optimize the classifier’s performance. The training is also performed with a cross-entropy loss function [38], optimized thought a stochastic gradient descent (SGD) technique. The adopted momentum is equal to 0.09 while the fixed decay is $1\times 10^{-6}$. The learning performances are also improved by configuring the SGD into all experiments with Nesterov accelerated gradient (NAG) correction (this allows to avoid excessive changes in the parameter space) [39].

## 4. Validation and Assessment

### 4.1. Dataset Construction

The dataset selected in this work is the public dataset acquired by the University of Glasgow (http://researchdata.gla.ac.uk/848/) containing C-band (carrier 5.8 GHz) micro-Doppler signatures of different human activities performed by different subjects in different environments [40]. The dataset has been acquired from individuals of male and female sex, left and right handed, aged between 21 and 98 years old and with an height interval between 149–198 cm. Each subject in the dataset performs the following activities 3 times: walking back and forth, sitting down on a chair, standing up, bending to pick up an object, drinking from a cup or glass and in some cases falls were also simulated. In this work only the acquisitions containing the subjects walking back and forth were considered, thus all the other activities were discarded and are not considered in this work. Compared with the dataset used in [20], this dataset is more challenging as presents data acquired with a lower carrier frequency (5.8 GHz vs. 77 GHz used in [20]) from a large number of subjects and in different environments. The total number of different individuas is 106 with a total number of 318 observations available from the various walking sessions. For each activity a spectrogram computed according to Equation (3) is obtained using three different Hamming windows of 0.5, 1 and 2 millisecond window with 98% overlap. The 3 different window sizes will be used in different testing setups to assess the influence of the time-frequency resolution trade-off. The MD signal is finally re-organized into windows with a length of 25.5 s of data with an overlap of 1 s, for the generation of both the validation and test set.

### 4.2. Experimental Settings

The proposed experiment aims to evaluate the effectiveness of our approach to identify a single walker with respect to the other ones. To this aim, the performances of the proposed classifier are evaluated by computing its precision, recall, F1, Accuracy, and Area Under Curve (AUC) [41] on a real dataset suitable for the feature model described in Section 3. In the assessment step, the best combination of the parameters reported in Table 2 is computed exploiting a Sequential Bayesian Model-based Optimization (SBMO) approach implemented by using a Tree Parzen Estimator (TPE) algorithm [42]. Table 2 lists the considered hyperparameters and their evaluated ranges. As an activation function, the ReLU is evaluated since it is widely adopted. However, it is known that the ReLU activation function suffers from the so-called “dead” units problem. The dying ReLU problem happens if the input becomes too large causing the gradient to update the weights in a way that the summation, and thus the input, will always be less than zero for the remaining of the entire training session. Since ReLU is defined as g(x) = max(0, x), if the input is always less than zero, it could just as well have been g(x) = 0. But since the gradient of a constant function is still zero, the weights won’t be updated anymore leading to the neuron effectively dying being trapped in a bad local minimum. For this reason, in this study also Swish and Mish are activation functions are also evaluated. However, they are recently proposed [36,43] since they are not affected by the “dead neurons” issue and give better performance in the case of the vanishing gradient problem.

For the network size, two levels (small and medium) are evaluated. The small size network has a maximum of 1.5 mln of learning parameters whereas a medium network has a number of parameters greater than 1.5 mln and lower of 7 mln. Looking at the learning rate, we consider a range between 5 and 15. These values are normalized with respect to the used optimization algorithm. For example, when the SGD optimizer is used, the learning rate ranges between 0.09 and 0.12.

The table also reports the evaluated number of layers (from 6 to 9) and the batch size. For the last one, three standard and widely used sizes (64, 128, and 256) are evaluated. Notice that for the batch size we observed that when it is greater than 256 the training process became less stable (this also influence the accuracy result).

Moeover, to minimize the loss, the three evaluated optimization algorithms are: the Stochastic Gradient Descent (SGD) [44], RmsProp [45], Nadam [45]. Finally in the table also the considered window sizes (64, 128 and 256) for the spectrogram computation. These correspond to the 0.5, 1 and 2 milliseconds time windows.

In the proposed experiments, the SGD is integrated with the Nesterov Accelerated Gradient (NAG) correction to reduce the possible changes in the parameter space [39].

The proposed experiments also allow evaluating the impact of the number of identified targets (walkers) on the classifier performance. To this aim, we evaluate the classifier performance when different numbers of subjects (10, 50, 100) are used for the analysis.

The neural network classifier is implemented by using PyTorch 1.4 deep learning framework and the training is performed on a machine with two Intel (R) Core (TM) i9 CPU 4.30 GHZ, 64 GB of RAM, and four Nvidia Titan XP.

### 4.3. Results and Discussion

Table 3 reports for the classifier performance at the best hyperparameters combinations when the training is performed respectively with a different number of targets (10, 50, 100). Looking at the 100-targets TCN classifier, we notice that the best accuracy (0.89) is reached when the temporal window size (WS) is of 128 seconds. The corresponding hyperparameters configuration provides Mish as activation function (AF), Nadam as optimization algorithms (OA), a large network size (NS), a batch size (BS) of 16, nine hidden layers (NL) and was trained by with learning rate (LR) equal to 0.15.

**Figure 7:**
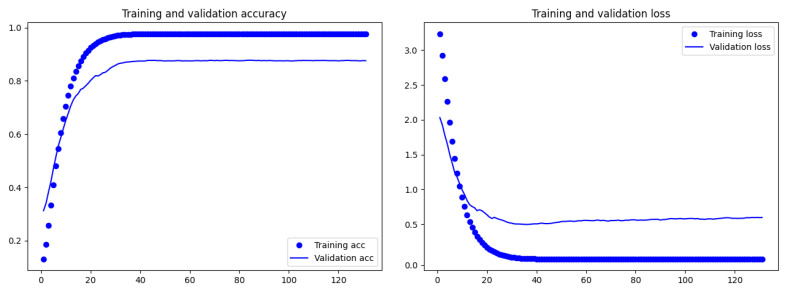
Training and validation accuracy and loss by epochs for the best 100-targets TCN classifier.

Figure 7 reports for the best 100-targets TCN classifier, the training and loss accuracy at the increasing of the epochs. The figure highlights that that accuracy and loss became stable starting from 40 epochs.

Table 4 also reports the performance of the TCN for different number of targets compared to the best standard classifiers. Specifically, we compared the proposed architecture with the state of the art models used for similar tasks (i.e., VGG16 and VGG19 [46], RESNET [47], and the standard CNN2D). The table shows that in all the cases the TCN gives best F1 and AUC.

Finally, we also evaluated the impact of the number of targets used for the classifier’s training on its performances.

Starting from Table 3, it is clear that the classifier performances get worse at the increasing of the number of targets used for the training. However, the table also shows that in all the cases the obtained performances are never less than 0.849 (it is the hyperparameters combination describe in the last row of the table). Looking to Table 4, it also clear that in all the tested classifiers the performances are similarly influenced by the number of targets used for the training. These results, for the CNN classifier, are also confirmed by the findings described in [26].

**Figure 8:**
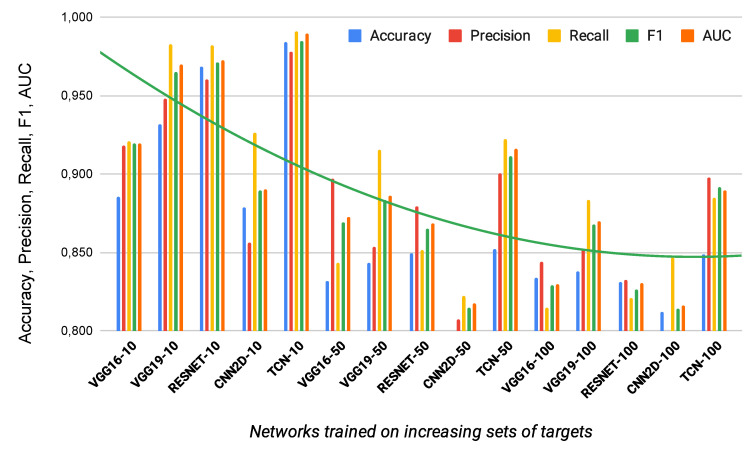
Performance of deep neural network for increasing number of identified targets.

As we can see from Figure 8, F1 decreases when the number of Targets increases but the proposed network (TCN) for a given number of detected users is always the best.

**Figure 9:**
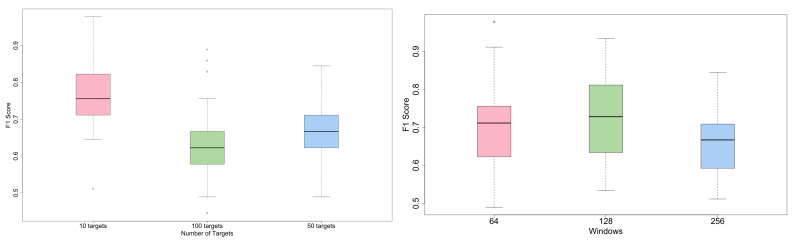
F1 score of the TCN classifier trained with different number of targets (**left**) and different windows (**right**).

Finally, Figure 9 (left side) show the different F1 scores of the TCN classifiers by the changing targets numbers. Similarly, the figure (right side) also shows that the best F1 score is generally obtained for the window size of 128. The figure confirms and generalizes the above considerations.

From the point of view of the performance of the end-to-end system, it is worth noting that the inference times are very short (in the order of milliseconds for a single image). For this reason the system can be implemented to operate in real time with the largest computational cost residing in the computation of the STFT that is then sent as input to the trained neural network.

## 5. Conclusions

This study introduces an approach based on a TCN classifier and a set of micro-Doppler features. The approach aims to perform gait recognition using the data extracted by a low-cost low-power FMCW radar. The accuracy of the classifier is evaluated on a real dataset acquired by the University of Glasgow. The dataset contains data gathered from walking sessions involving a total number of 106 different individuals a total number of 318 observations available from the various walking sessions. The performance of the TCN classifier is evaluated considering different hyper-parameters combinations, different window sizes, and different numbers of targets. In all the cases, the results show the greater performance of the TCN classifier with respect to other baseline ones (CNN2D, RESNET, VGG16, and VGG19).

Finally, we also observed that the number of targets used for the classifier’s training and the window size influence the classifier performances. However, the classifier performances get worse with an increase in the number of targets used for the training. Moreover, the best F1 score is obtained when the windows size is 128, probably because that is the best tradeoff between time and frequency resolution. The results confirm that 8-layer TCN networks, augmented with hierarchical attention layers, are suitable for identification of up to a hundred of walkers with good quality classification performances (F1 = 0.9).

## Figures and Tables

**Table 1:** List of used symbols and their meanings.

$R_{0}$	range from center of (x’,y’,z’) to radar in (x,y,z)
$r_{t}$	range from point-target to radar in (x,y,z)
$D_{v}$	range from point-target to center of (x’,y’,z’)
$f_{v}$	vibration frequency
α	azimuth angle of the center of (x’,y’,z’)
β	elevation angle of center of (x’,y’,z’)
$\alpha_{p}$	azimuth angle of *P* relative to center of (x’,y’,z’)
$\beta_{p}$	elevation angle of *P* relative to center of (x’,y’,z’)

**Table 2:** Hyper-parameters Optimization space.

Hyperparameters	Acronym	Optimized Ranges and Sets
Activation function	AF	{ReLU, Swish, Mish}
Batch size	BS	{ 32, 64, 128, 256 }
Learning rate	LR	[0.09, 0.15]
Network size	NS	{Small, Medium, Large}
Number of layers	L	{ 6, 7, 8, 9 }
Optimization algorithm	OA	{SGD, Nadam, RMSprop}
Window size	WS	{64, 128, 256}

**Table 3:** Hyperparameters optimization summary: best three.

Targets	AF	NS	LR	NL	BS	OA	WS	Precision	Recall	F1	Accuracy	AUC
	**Swish**	**Medium**	**0.12**	**6**	**64**	**Nadam**	**128**	**0.984**	**0.978**	**0.991**	**0.984**	**0.989**
10	ReLu	Medium	0.15	6	128	SGD	128	0.970	0.963	0.977	0.968	0.971
	ReLu	Small	0.09	8	128	SGD	64	0.952	0.952	0.960	0.950	0.961
	**Mish**	**Large**	**0.12**	**7**	**32**	**Nadam**	**128**	**0.852**	**0.901**	**0.922**	**0.911**	**0.916**
50	Swish	Medium	0.10	8	32	SGD	128	0.798	0.839	0.892	0.871	0.895
	Swish	Medium	0.12	8	64	SGD	128	0.773	0.811	0.872	0,825	0.859
	**Mish**	**Large**	**0.15**	**9**	**16**	**Nadam**	**128**	**0.849**	**0.898**	**0.885**	**0.891**	**0.890**
100	Mish	Large	0.14	9	32	RMSProp	128	0.830	0.851	0.838	0.862	0.871
	Mish	Large	0.15	9	32	SGD	256	0.789	0.823	0.788	0.838	0.849

**Table 4:** Performance comparison of the TCN classifiers with baseline methods (CNN2D, RESNET, VGG16, VGG19).

Target	Network	Accuracy	Precision	Recall	F1	AUC
	VGG16	0,886	0.918	0.921	0.919	0.920
	VGG19	0.932	0.948	0.983	0.965	0.969
10	RESNET	0.969	0.960	0.982	0.971	0.973
	CNN2D	0.879	0.856	0.926	0.890	0.890
	**TCN**	**0.984**	**0.978**	**0.991**	**0.984**	**0.989**
	VGG16	0.832	0.897	0.843	0.869	0.872
	VGG19	0.843	0.853	0.915	0.883	0.886
50	RESNET	0.850	0.880	0.851	0.865	0.868
	CNN2D	0.766	0.807	0.822	0.815	0.817
	**TCN**	**0.852**	**0.901**	**0.922**	**0.911**	**0.916**
	VGG16	0.834	0.844	0.815	0.829	0.830
	VGG19	0.838	0.852	0.883	0.867	0.870
100	RESNET	0.831	0.832	0.821	0.827	0.831
	CNN2D	0.812	0.784	0.847	0.814	0.816
	**TCN**	**0.849**	**0.898**	**0.885**	**0.891**	**0.890**

## Data Availability

Not applicable.
